# Pseudogene UBE2MP1 derived transcript enhances *in vitro* cell proliferation and apoptosis resistance of hepatocellular carcinoma cells through miR-145-5p/RGS3 axis

**DOI:** 10.18632/aging.204319

**Published:** 2022-10-07

**Authors:** Fengjie Hao, Nan Wang, Honglian Gui, Yifan Zhang, Zhiyuan Wu, Junqing Wang

**Affiliations:** 1 Department of General Surgery, Ruijin Hospital, Shanghai Jiao Tong University School of Medicine, Shanghai 200025, People’s Republic of China; 2 Department of Infectious Disease, Ruijin Hospital, Shanghai Jiao Tong University School of Medicine, Shanghai 200025, People’s Republic of China; 3 Department of Interventional Radiology, Ruijin Hospital, Shanghai Jiao Tong University School of Medicine, Shanghai 200025, People’s Republic of China

**Keywords:** pseudogene, UBE2MP1, miR-145-5p/RGS3 axis, hepatocellular carcinoma, cell growth

## Abstract

Pseudogenes are barely transcribed at normal, while the anomalous transcripts of them are mostly regarded as long non-coding RNAs (lncRNAs), which play potential functions in human tumorigenicity and development. The exact effects of pseudogene-derived transcripts on hepatocellular carcinoma (HCC) are ambiguous. According to our previous research and constructed database on the HCC-related lncRNAs, we noticed that UBE2MP1 was transcriptionally activated in HCC as a pseudogene from the ubiquitin-conjugating enzyme member UBE2M. In this study, we validated the high expression of the UBE2MP1 transcript in HCC and its adverse correlation with dismal outcomes for the patients. UBE2MP1 depletion at the transcript level significantly impaired cell proliferation and apoptosis resistance in HCC cell lines. Notably, we discovered that the UBE2MP1 transcript shared a specific sequence, binding to the miR-145-5p seed region with a typical ceRNA effect. Simultaneously, we verified an axis of miR-145-5p/RGS3 in HCC cells, which promoted cell proliferation and apoptosis resistance with significance. And modulation of UE2MP1 could remarkably affect RGS3 expression and consequentially influence HCC cell growth *in vitro*. And combined with the rescue experiment modulating either miR-145-5p or RGS3 furtherly indicated UBE2MP1 as an upstream regulator of the axis in promoting HCC cell growth and maintenance. Thus, our findings provide new strategies for HCC prevention and individual treatment.

## INTRODUCTION

Hepatocellular carcinoma (HCC) results in severe tumor-related mortality and creates an urgent need for improving prognosis and long-term survival time for the patients [1, 2]. Targeted therapies are widely used in the clinical treatment of HCC, dependently or combined with other systematic therapies. However, the overall survival of the patients has not been accomplished to a satisfactory clinical endpoint [3]. It is important to develop more innovative therapeutic targets for the enrichment of practical HCC preventional and treating strategies.

As acknowledged, a large number of transcripts exist, which are categorized into different RNA species, generated from the human genome, and the majority of these transcriptional products lack the protein-coding capacity, called the non-coding RNAs (ncRNAs). Two major classes have been identified, the small ncRNAs with the composition of fewer than 200 nucleotides, represented by the miRNAs; and the long non-coding RNAs (lncRNAs), composed with over 200 nucleotides, covering the complex mechanisms of either transcriptional or post-transcriptional events [4, 5].

Due to the large number of lncRNAs and their localization in different intracellular compartments, these kinds of molecules influence various pathological or physiological functions in the cell by interacting with DNAs, RNAs, or proteins, and are involved in gene transcription, mRNA translation, protein modification, and the formation of RNA-protein or protein-protein complexes [6, 7].

Pseudogenes are special sequences of the DNA, generated through acquired mutation and duplication of their parental genes [8, 9]. Commonly, pseudogenes are barely transcribed because of the structural defects on the promoter, premature stop codon, or the occurrence of frameshift mutation [10]. Mostly, the pseudogene-derived transcripts are considered the lncRNAs with a length of over 200 nucleotides. Taking advantage of the acknowledged mechanism by which lncRNAs exert their complex functions, we can still make sense of the pseudogene-derived transcripts functions, for example, the miRNA decoy effect, or competitive endogenous RNA (ceRNA) effect.

On the other hand, the ceRNA effect is hypothesized based on the competitive interaction with the high affinity between miRNAs and the specific sequences in the lncRNAs. And this effect can abrogate the degradation of the targeted mRNAs induced by the specific miRNAs [11, 12]. A series of pseudogene-derived transcripts have been discovered to exert the ceRNA effect in human cancers and provide us with a new focus on the breakthroughs in the mechanism of HCC progress.

In this study, the pseudogene of Ubiquitin-conjugating enzyme E2M (UBE2M), named UBE2M pseudogene 1 (UBE2MP1) was noticed highly transcribed in HCC with significance. Our preliminary results from the *in vitro* experiments demonstrated a significant correlation between UBE2MP1 and the dismal features of the patients’ clinicopathological information. And depletion of the UBE2MP1 transcript in two HCC cell lines induced remarkable defection of cell proliferation and led to a high rate of cell apoptosis. We also discovered an axis of miR-145-5p/RGS3 (Regulator of G-protein signaling 3), which promotes HCC cell growth and maintenance. And intriguingly, we explored and discovered a specific sequence in the UBE2MP1 transcript, which shares a high affinity to bind the seed region of miR-145-5p in a molecular sponge way. The modulation of either miR-145-5p or RGS3 for rescue experiments could significantly recover the phenotypes of tumor cell growth and maintenance induced by UBE2MP1. We concluded and illustrated the tumor-promoting mechanism of UBE2MP1, independent of its parental genes, by modulating the miR-145-5p/RGS3 axis. And we prompt that these findings may provide innovative and hopeful targets for HCC control.

## MATERIALS AND METHODS

### Cell lines

Three typical HCC cell lines (Huh7, HepG2, and Hep3B) were recruited, and the normal human hepatic cell LO2 was used as control (Shanghai Institutes for Biological Sciences, Chinese Academy of Science, Shanghai, China)). All the cell lines were cultured by RPMI 1640, supplemented with 10% heat-inactivated fetal bovine serum (FBS), incubated at 37° C environment temperature, with 100 ug/ml streptomycin, and 100U/ml Penicillin in a humidified cell, with an atmosphere of 5% CO_2_. Specifically, for the transfected cells, a medium mixed with G418 (Santa Cruz Biotechnology, Inc; 400 μg/ml) was used for selection.

### Clinicopathological specimens

Nighty-three paired specimens including the tumor tissue and the adjacent non-cancerous liver tissues were collected from the patients diagnosed and conducted radical resection with no preoperative treatment, during 2016–2019, at the Department of General Surgery, Ruijin Hospital, Shanghai Jiao Tong University School of Medicine. The corresponding clinicopathologic parameters of the patients were obtained including gender, age, tumor size, number of lesions, grades et al. The informed consent was obtained, and the study was approved by the Ethics Committee of Ruijin Hospital, Shanghai Jiao Tong University School of Medicine.

### Datasets preparation

The gene expression data for 110 normal liver samples from the GTEx and TCGA along with clinical information for 369 liver tumors and 50 normal samples from the UCSC Xena database were intensively explored by using the random walk-based multi-graphic (RWMG) model algorithm developed by our team [13]. In brief, the RWMG model was developed from the biophysical interaction networks and the co-expression profiles within a single analytical framework, by which it integrates sophisticated biological connections among lncRNA targets, including the transcription factors (TF), microRNAs (miRNAs), and the alternative splice factors. This model presents flexible and scalable characteristics in ranking a subset of lncRNAs based on the literature survey. The RWMG model provides more accurate results than the previous network-based algorithms defined as the ‘shortest path’ or the conventional random walk algorithms and can avoid the ‘noise’ from the dimensional heterogenicity of the data. And the related and comprehensive information of the involved pseudogene-derived lncRNAs was described in the mentioned LCLE online. The starBase datasets (https://starbase.sysu.edu.cn/) and the dreamBase (https://rna.sysu.edu.cn/dreamBase/) datasets were used for providing supplementary information on the expression and relationship of the candidate genes in this study.

### RT-qPCR assay and immunohistochemistry assay

For RNA isolation, from either tissues or cells, conducted according to the instruction of the TRIzol reagent (Invitrogen, MA, USA). The first-strand cDNA was synthesized via High-Capacity cDNA Reverse Transcription Kit (ABI, NY, USA). All the primers were synthesized (Jike Biotech Company, Shanghai, China) (Supplementary Table 1). Real-time quantitative polymerase chain reaction (RT-qPCR) was operated following the TaqMan Gene Expression Assays protocol (ABI, NY, USA). The relative quantification of RNA in cell lines was normalized using GAPDH by the 2*−*Δ*CT* method. And, the relative quantification of miR-145-5p in tissue specimens and cell lines was measured by using the mirVANATM miRNA Isolation Kit (ABI, MA USA). The PCR program was set as follows: 95° C for 10 min, followed by 35 cycles of 95° C for 15s, 60° C for the 30s, and 72° C for 45s.

Antibody against RGS3 was prepared (Abcam, MA, USA). The immunohistochemistry assay complied with our previously described methods [14]. The protein expression levels detected by IHC were assigned to two experienced pathologists independently for blind examination and were separated into two groups by staining intensity grade: no to low staining (0–1+) and moderate to high staining (2+–3+).

### Preparation of the plasmid and cell transfection

The lentiviral vectors pLKO.1 (Addgene, Cambridge, MA, USA) containing shRNA were transfected into cultured HepG2 and Hep3B cells at exponential phase (JIKE Biochemistry, Shanghai, China) for suppressing the expression of UBE2MP1 transcript. Meanwhile, the control vectors were set up. The transfected cells were selected by using a medium mixed with G418 (Santa Cruz Biotechnology, Inc; 400 μg/ml). The mimic was used to transfect HCC cells for ectopically introducing miR-145-5p (HepG2/miR-145-5p; Hep3B/miR-145-5p), and the negative controls (HepG2/NigmiR; Hep3B/NigmiR) were set. And the rescue experiment by knock-out miR-145-5p was set up by using the siRNA method by using the protocol and siRNA vector tools designed by Jike Biotech Company, (Shanghai, China), and the validation was implemented by the RT-qPCR assay. The lentiviral vector pLV (Addgene, Cambridge, MA, USA) was applied for ectopically re-expressing either RGS3 (pLV- RGS3) for the rescue experiments, and the pLV-Null was set as control.

### Cell proliferation and cell cycle detection

The treated HCC cells (1x10^6^) were cultured in 96-well microtiter plates triplicated and incubated at an atmosphere of 5% CO_2_ and 37° C for 5 days. Microplate computer software (Bio-Rad Laboratories, Inc., Hercules, CA, USA) was applied for measuring the OD following the Cell Counting Kit-8 (CCK-8) assay kit protocol (Dojindo, Tokyo, Japan). Then, we plotted the cell proliferation curves. Meanwhile, the cells were treated with ethanol fixation, followed by RNase A treatment and propidium iodide staining. Flow cytometry detection was carried out using FACSCalibur (Becton-Dickinson, Franklin Lakes, NJ, USA) for quantifying cell populations at the G0/G1, S, and G2/M phases, and ModFit software e (Becton-Dickinson) was used. The debris and fixation artifacts of the cells were excluded.

### Cell apoptosis analysis

Cell apoptosis rate was calculated by using PE-Annexin V Apoptosis Detection Kit I (BD Pharmingen, USA) following the instructions. Transfected cells were resuspended in the concentration of 1×10^6^ cells/ml by the 1×Binding Buffer. 5μl of FITC and 5μl of PI were added into 100μl of the cell suspension, followed by a 15 minutes incubation in darkness, added with 400μl×Binding Buffer. The apoptosis rate was calculated through flow cytometry (Becton-Dickinson). Both Annexin V-FITC-positive and PI-negative cells were considered apoptosis cells.

### The dual-luciferase reporter assay

By using the online tools of microcosm (http://mirecords.biolead.org) and dreamBase (http://rna.sysu.edu.cn/dreamBase/), miR-145-5p was respectively predicted directly binding to the sequences of either the 3’-untranslated region (3’-UTR) of RGS3 mRNA or the UBE2MP1 transcript. Both of the sequences above were intercepted for the 202 bp sections along with the corresponding mutative ones for further detection by the dual-luciferase reporter assay. (Supplementary Table 2). The sequences were respectively cloned into the pMIR-Report luciferase vector, which contains firefly luciferase, and the pRL-TK vector luciferase was set as control (Promega, Madison, WI, USA). These two sets of vectors were co-transfected into both HepG2 and Hep3B cells transfected miR-145a-5p mimics or the control ones. The luciferase activity was measured via the Dual-Glo Luciferase assay system (Promega) 48 hours after the transfection.

### Statistical analysis

Statistical analysis was carried out by using SPSS 20.0. *P*-values were calculated using an unpaired Student's *t*-test and Fisher's exact test and the one-way ANOVA as the statistical methods. Differences were considered statistically significant at *P*-values < 0.05.

## RESULTS

### Pseudogene UBE2MP1 was anomalously transcripted in HCC cell lines and tissues

**Figure 1:**
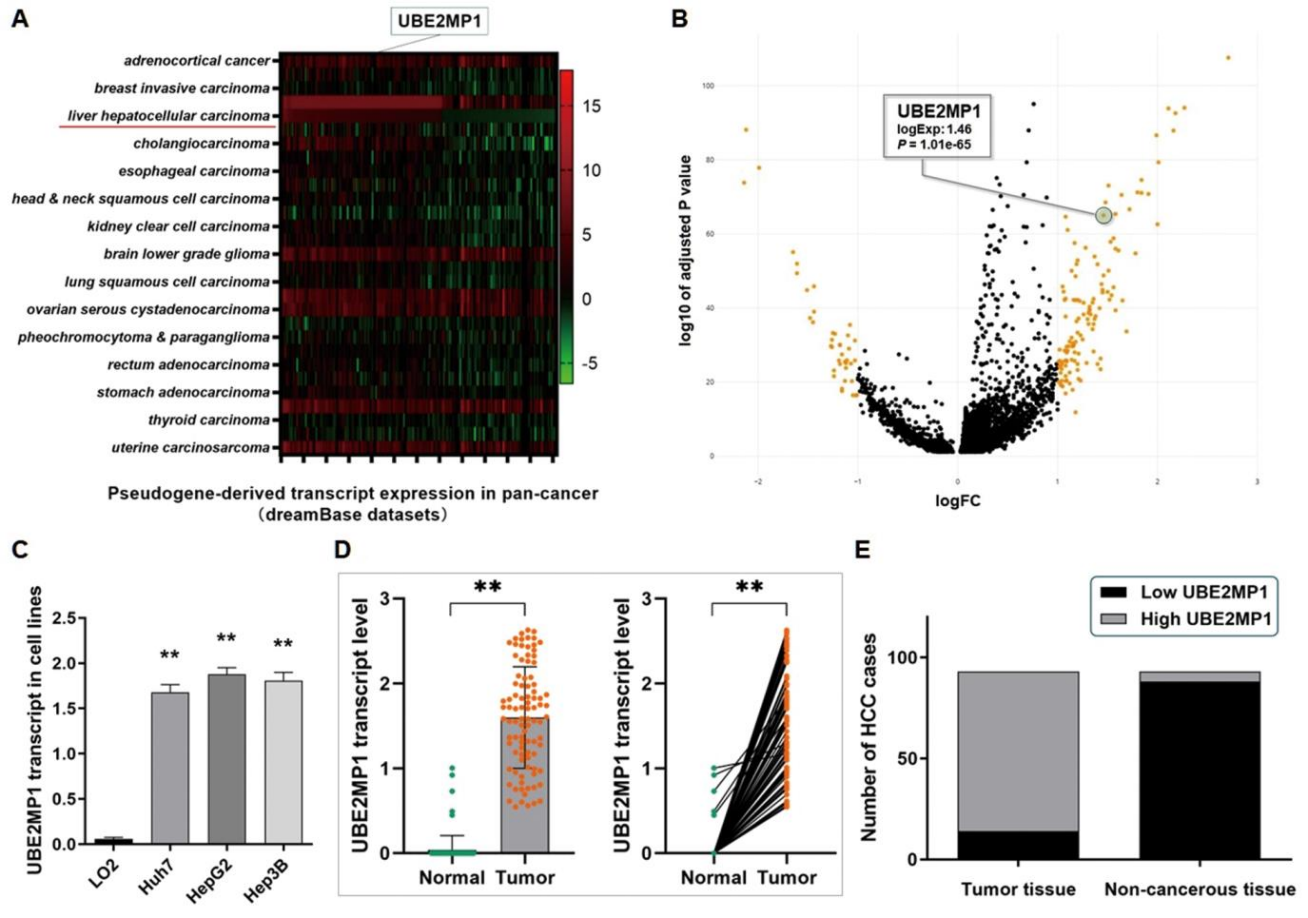
**Pseudogene UBE2MP1 was anomalously transcripted in HCC cell lines and tissues.** (**A**) Analysis of the dreamBase datasets. The heatmap indicated the expression profile of the transcript of UBE2MP1 in pan-cancer. (**B**) Analysis of the data from the LCLE. Pseudogene UBE2MP1 is transcriptionally activated and presents a high expression in HCC. (**C**) RT-qPCR assay demonstrated a significantly high expression of UBE2MP1 transcript in three HCC cell lines, in comparison with the control LO2 cells barely transcribed this pseudogene (**P<0.01). (**D**) RT-qPCR assay was conducted on 93 real patients’ specimens. The UBE2MP1 transcript was significantly highly expressed in tumor tissues, and only very few detectable UBE2MP1 transcript was found in the non-cancerous tissues (**P<0.01). (**E**) Statistic of the number of cases concerning the expression of UBE2MP1 transcript in HCC specimens. UBE2MP1 transcript is detectable and highly expressed in most of the tumor tissues (79/93), and was not detected in most of the adjacent non-cancerous tissues (88/93) (*P*<0.01).

The exploration and analysis of the datasets from the dreamBase databases indicated a universal transcription of UBE2MP1 in either pan-cancers or HCC (Figure 1A). On basis of a random walk-based multi-graphic (RWMG) model algorithm we developed, a series of pseudogenes generating lncRNA-like transcripts in HCC were screened out from the analysis tools of the Liver Cancer lncRNA Explore (LCLE) online tool (https://datasciences123.shinyapps.io/LCLE/) [13], and UBE2MP1 and the volcano plot that presents the expression profile of the pseudogene transcript indicated a similar result (Figure 1B). As we observed, merely no detectable UBE2MP1 transcript was found in the normal organs and tissues, including the liver. Whereas, the up-regulation of the UBE2MP1 transcript was detected in different tumor tissues. Especially for HCC, the remarkable elevation of the UBE2MP1 transcript was observed.

The UBE2MP1 transcript expression in three HCC cell lines (Huh7, HepG2, and Hep3B) was detected by RT-qPCR assay. The level of UBE2MP1 transcript in three HCC cell lines is significantly higher than in the control LO2 cells (Figure 1C). For the real patients’ specimens from our medical center, a high level of UBE2MP1 transcript was detected in HCC tumor samples, and the adjacent non-cancerous liver tissues presented nearly no sign of UBE2MP1 transcription. As Figure 1D, 1E show, the UBE2MP1 transcript is detectable in all of the tumor specimens, and 84.95% (79/93) of the HCC specimens presented a high level of UBE2MP1 transcript expression, and the rest 15.05% (14/93) presented a relatively lower level UBE2MP1 expression. On the contrary, only 5.38% (5/93) of the adjacent non-cancerous liver tissues presented detectable but low expression of UBE2MP1. Simultaneously, we also validated the high expression of the parental gene UBE2M by RT-qPCR assay and the IHC assay in the real patient specimens and cell lines, which suggests a co-expression inclination between these two genes (Supplementary Figure 1).

### Transcription of UBE2MP1 is correlated with the HCC patients’ clinicopathologic features

The correlation between the transcription of UBE2MP1 in HCC and the clinicopathologic features of the 93 real HCC patients was analyzed statistically by Fisher's exact test and the one-way ANOVA. There is no significant correlation between UBE2MP1 and the patient’s age, gender, and virus control status. Whereas, UBE2MP1 transcription shows an adverse relationship with the tumor size (*P*<0.05), serum Alpha-fetoprotein (AFP) quantity (*P*<0.05), more advanced TNM stages (*P*<0.05), tumor microsatellite formation (*P*<0.05), invasion of venous (*P*<0.05), and liver cirrhosis stages (*P*<0.05) (Table 1). The findings here strongly suggest that the UBE2MP1 transcript plays a positive role in HCC development.

**Table 1:** Correlation between UBE2MP1 transcript and clinicopathological features in 93 HCC specimens.

**Clinicopathologic parameters**	**UBE2MP1 transcript**		P*
	**Low (n=14)**	**High(n=79)**	
Age (years)			
≤50	8	54	0.540
>50	6	25	
Gender			
Male	10	41	0.246
Female	4	38	
Diameter (cm)			
≤5	12	31	0.003
>5	2	48	
TNM stage			
I~II	12	30	0.001
III~IV	2	49	
Tumor encapsulation			
Absent	9	31	0.141
Present	5	48	
Tumor microsatellite formation			
Absent	12	29	9.0e-04
Present	2	50	
Venous invasion			
No	10	22	0.004
Yes	4	57	
HBsAg			
Negative	5	11	0.061
Positive	9	68	
AFP(ng/ml)			
≤400	12	12	1.0e-04
>400	2	67	
Cirrhosis			
Absent	4	8	0.079
Present	10	71	

*P<0.05.

UBE2MP1 transcript levels, associated with clinicopathologic features in 93 HCC patients, including age, gender, tumor size, tumor stage (AJCC), tumor encapsulation, tumor microsatellite formation, vein invasion, HBsAg status, AFP level, and liver cirrhosis. Statistically, significance was assessed by Fisher’s exact test.

### UBE2MP1 depletion impairs cell proliferation and arrests cell cycles in HCC

**Figure 2:**
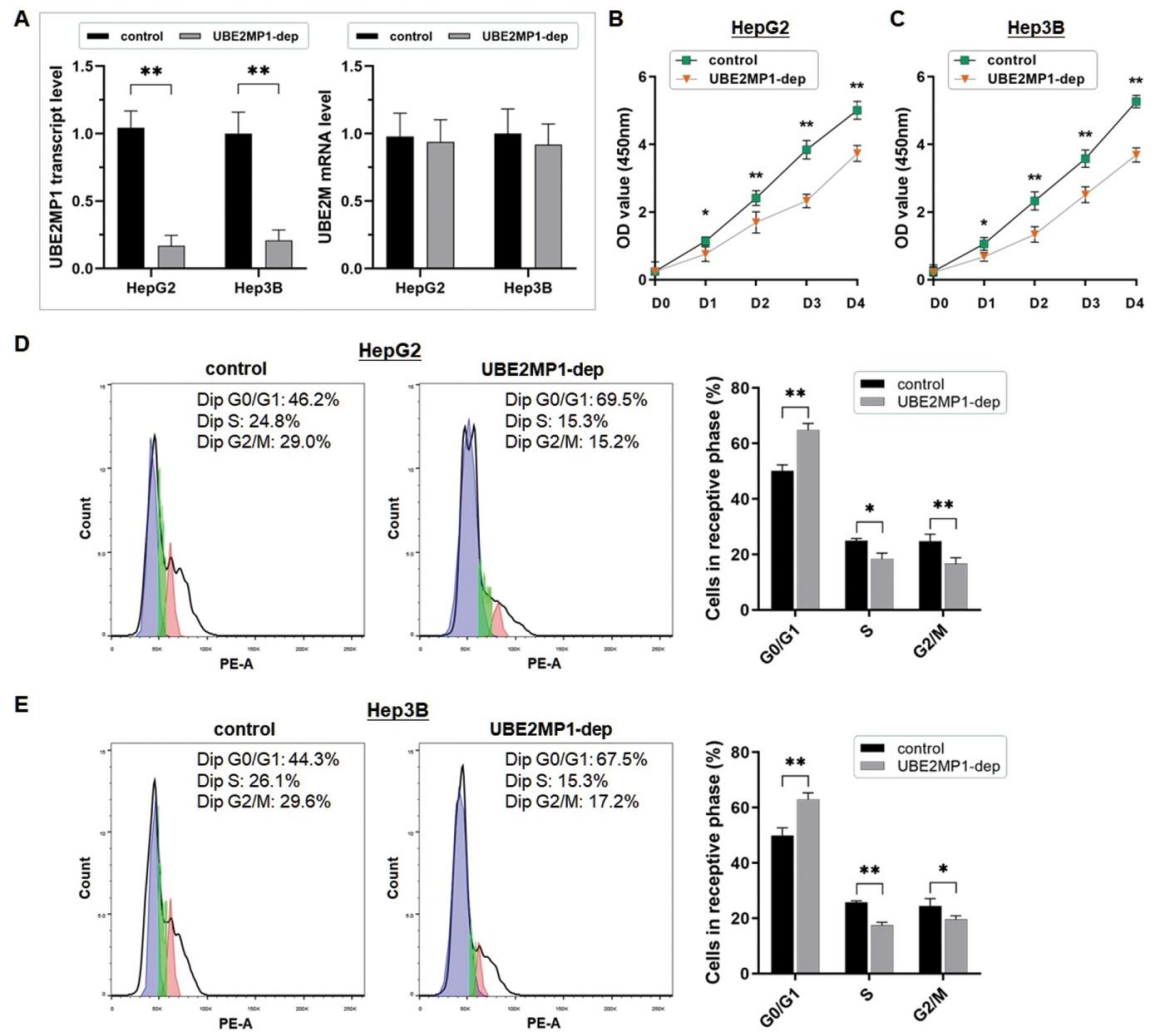
**UBE2MP1 Depletion impairs cell proliferation and arrests cell cycles in HCC.** (**A**) UBE2MP1 depletion was conducted in HepG2 and Hep3B cells by using shRNA transfection. The RT-qPCR assay demonstrated the significant abrogation of UBE2MP1 transcript in the two treated cell lines, and no significant change of the parental gene UBE2M was induced (**P<0.01). (**B**, **C**) The CCK8 assay was conducted to describe cell proliferation status. The cell proliferation in both the two cell lines was significantly suppressed by abrogating the UBE2MP1 transcript (*P<0.05; **P<0.01). (**D**, **E**) Flow cytometry was used for analyzing the cell cycle distribution. And the representative histograms were shown. The cell cycle of both HepG2 and Hep3B cells was significantly arrested in G0/G1 phase by depleting the UBE2MP1 transcript. The results are means of three independent experiments ±SD. (*P< 0.05, **P< 0.01).

The RT-qPCR assay was conducted to validate the effect on UBE2MP1 depletion, in both HepG2 and Hep3B cells, and the expression of the parental gene was not affected (Figure 2A). The cell proliferation was significantly suppressed in both the two cell lines by abrogating the UBE2MP1 transcript (unpaired Student's *t*-test; *P<0.05; **P<0.01) (Figure 2B, 2C).

The flow cytometric analysis showed a significant cell cycle arrest at the G0/G1 phases in the HCC cells when UBE2MP1 was depleted (Figure 2D, 2E). The percentage of the HepG2 and Hep3B cells in the G0/G1 phase increased respectively from 50.2% to 64.8% (*P*<0.01) and from 49.9% to 63.0% (*P*<0.01) (Student's *t*-test). Meanwhile, the obvious percentage decrease was observed in the S phase (HepG2: from 25.0% to 18.5%, *P*<0.05; Hep3B: from 25.7% to 17.5%, *P*<0.01) and the G2/M phase (HepG2: from 24.8% to 16.7%, *P*<0.01; Hep3B: from 24.4% to 19.5%, *P*<0.05).

### Depletion of UBE2MP1 enhances HCC cell apoptosis

**Figure 3:**
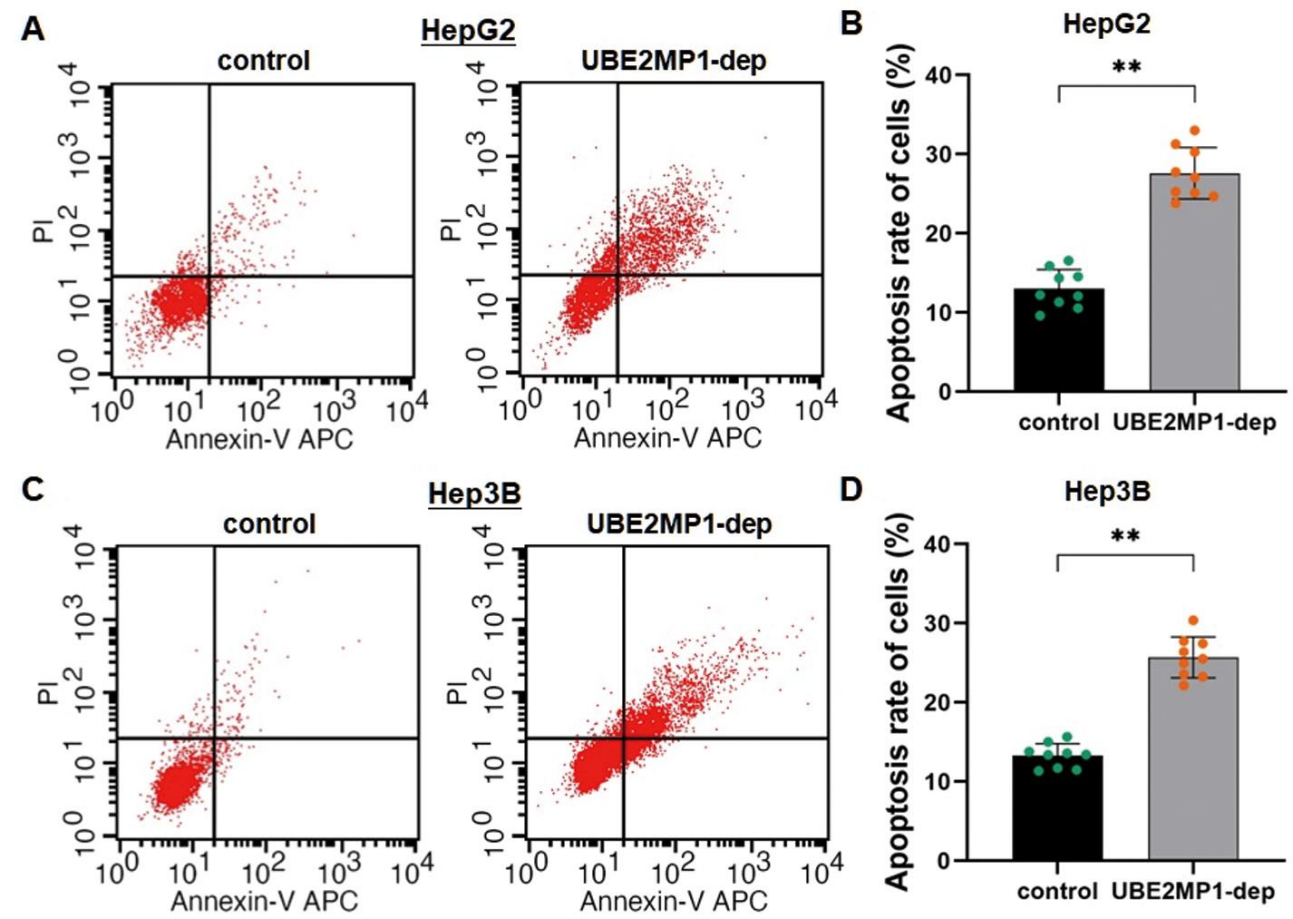
**Depletion of UBE2MP1 enhances HCC cell apoptosis.** (**A**) Cell apoptosis was detected by flow cytometry. The representative histograms demonstrate the apoptosis rate in HepG2 cells. (**B**) The counting number of the apoptotic cells was significantly increased in HepG2 cells by depleting UBE2MP1 (**P<0.01). The results are means of three independent experiments ±SD. (**C**) The representative histograms demonstrate the apoptosis rate in Hep3B cells. (**D**) The counting number of the apoptotic cells was significantly increased in HepG2 cells by depleting UBE2MP1 (**P<0.01). The results are means of three independent experiments ±SD.

The flow cytometric analysis was applied for calculating the statuses of cell apoptosis by an unpaired Student's *t*-test. The cell apoptosis rates in both HepG2 and Hep3B cells were significantly increased (HepG2: from 13.01% to 27.59%, *P*<0.01; Hep3B: from 13.27% to 25.71%, *P*<0.01) following the depletion of UBE2MP. This finding suggested that the transcription of UBE2MP1 in HCC could enhance cell maintenance by inhibiting cell apoptosis (Figure 3A–3D).

### UBE2MP1 transcript sponges miR-145-5p and was positively correlated with RGS3

**Figure 4:**
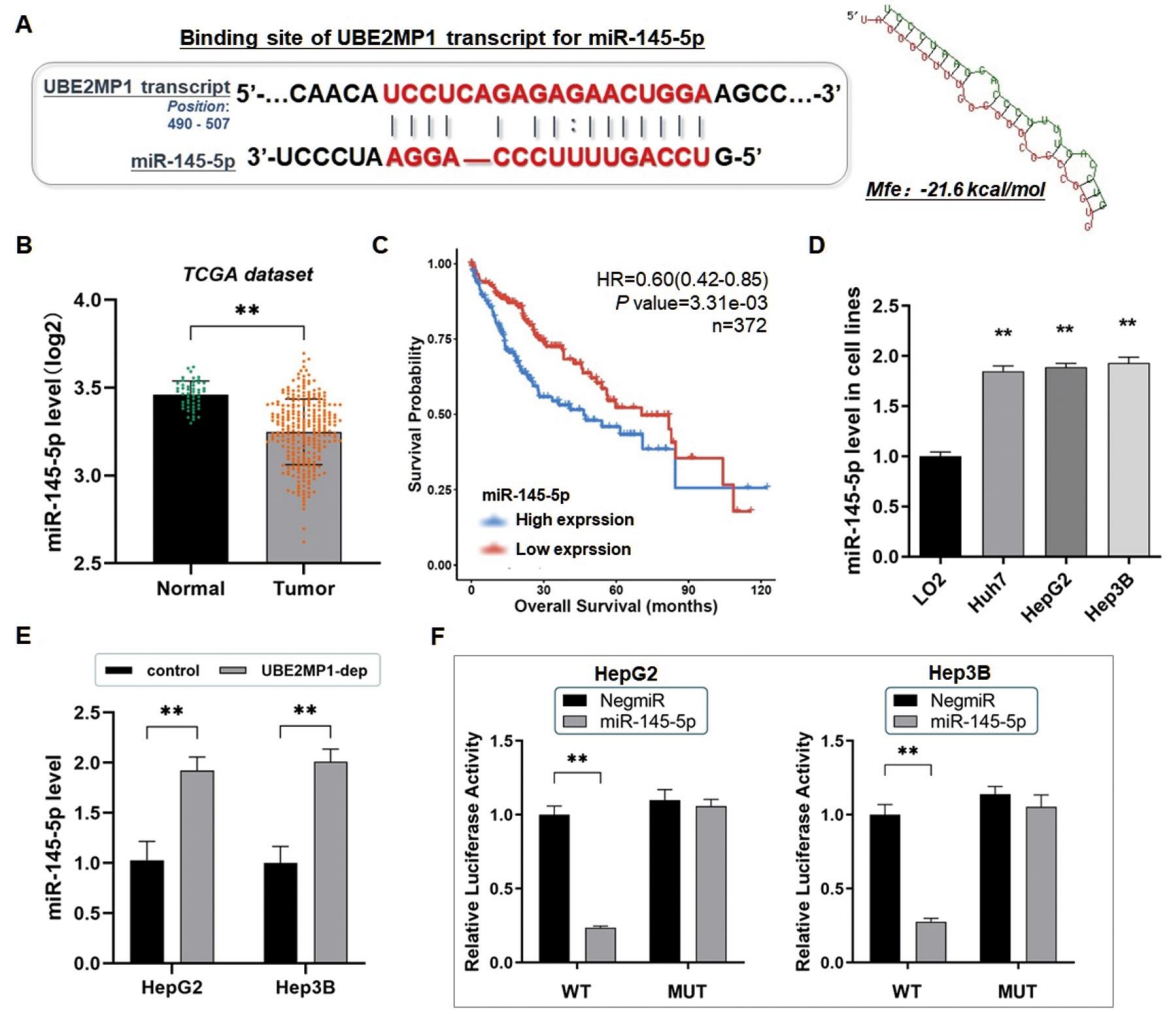
**UBE2MP1 transcript sponges miR-145-5p and was positively correlated with RGS3.** (**A**) Predicted binding sequence of UBE2MP1 transcript with the seed sequence of miR-145-5p. The minimum free energy (Mfe) hybridization is calculated as: -21.6 kcal/mol. The comprehensive expression profile of miR-145-5p in pan-cancers (**P<0.001). (**B**) The expression profile of miR-145-5p in HCC through analysis of the dataset from the TCGA database (**P<0.01). (**C**) Exploration of the TCGA datasets indicated a significant decline of miR-145-5p in HCC associated with shortened overall survival. (**D**) The RT-qPCR assay demonstrated a significant decrease of miR-145-5p in three HCC cell lines, in comparison with the control LO2 cells (**P<0.01). (**E**) The expression of miR-145-5p in both HepG2 and Hep3B cells was significantly elevated by depleting UBE2MP1 (**P<0.01). (**F**) The dual-luciferase reporter assay verified the direct interaction of UBE2MP1 to miR-145-5p, as a molecular sponge (**P<0.01).

We explored the UBE2MP1 transcript and found that a sequence from 490 bp to 507 bp to the 3’ end of the UBE2MP1 transcript is probably a specific binding site that matches the seed region of miR-145-5p, which prompts a ceRNA effect of UBE2MP1 on miR-145a-5p (The minimum free energy, Mfe: -21.6 kcal/mol) (Figure 4A). The exploration of TCGA datasets also indicated a significant decline of miR-145-5p in HCC and was associated with poor clinical survival outcomes (Figure 4B, 4C). And according to the RT-qPCR assay, miR-145-5p was expressed at a relatively lower level in the HCC cell lines compared with the control LO2 cells (Figure 4D). Moreover, the expression of miR-145-5p was consequentially increased after the depletion of UBE2MP1 in the two HCC cell lines (Figure 4E).

Based on this, we constructed the mutated binding site on UBE2MP1, and we carried out the dual-luciferase reporter assay to verify the direct interaction between UBE2MP1 and miR-145-5p. As discovered, the luciferase signal in either HepG2 or Hep3B cells transfected with miR-145-5p mimics was decreased significantly, after the transfection of the UBE2MP1/pMIR/WT vector, in comparison with the control ones. On the contrary, the transfection of the UBE2MP1/pMIR/MUT vector did not induce signal changes with significance (Figure 4F). Thus, the findings convincingly illustrated the ceRNA effect of UBE2MP1 on sponging miR-145-5p.

### RGS3 mRNA is targeted by miR-145-5p post-transcriptionally

**Figure 5:**
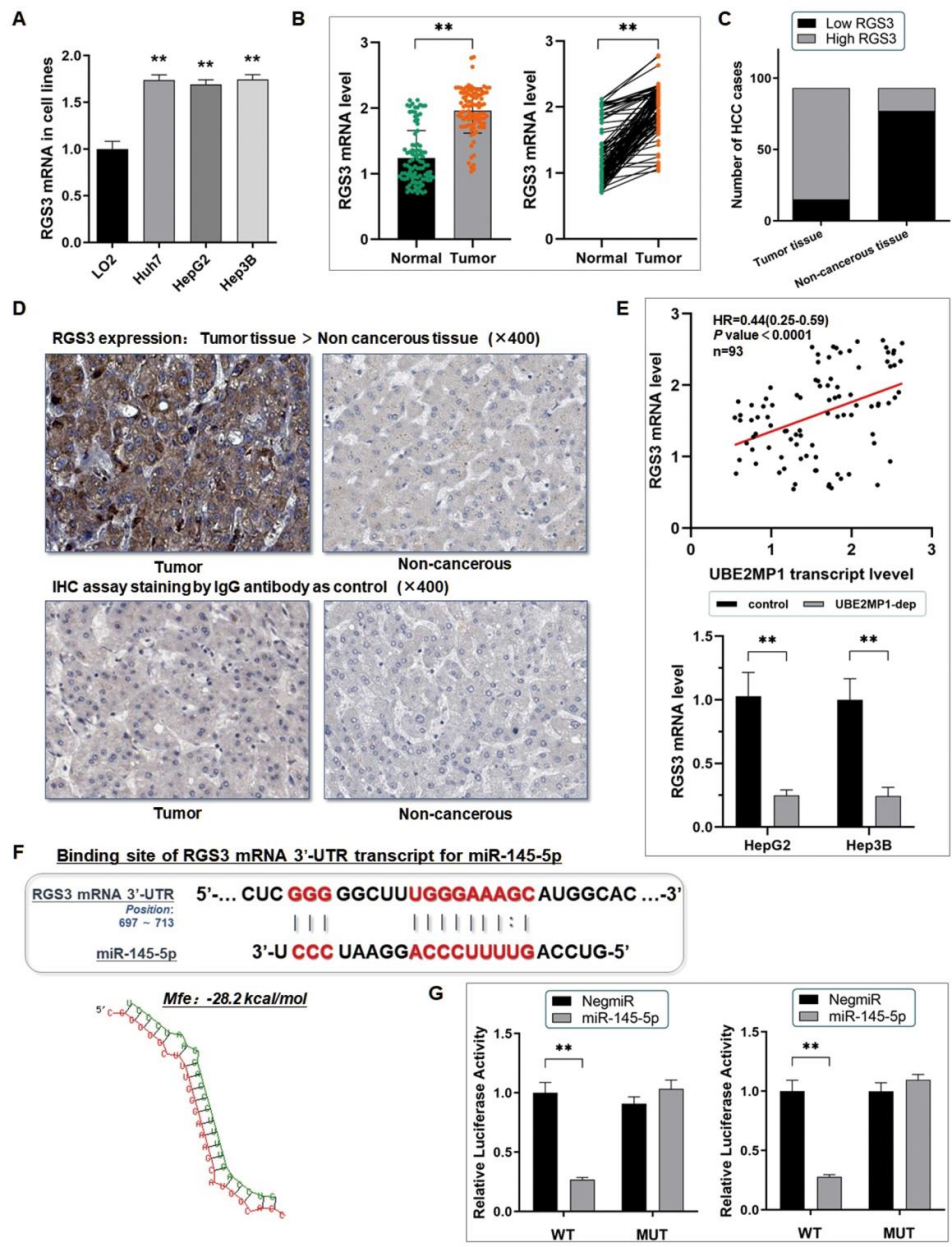
**RGS3 mRNA is targeted by miR-145-5p post-transcriptionally.** (**A**) The RT-qPCR assay demonstrated a significant up-regulation of RGS3 mRNA in three HCC cell lines, in comparison with the control LO2 cells (**P<0.01). (**B**) RT-qPCR assay was conducted on 93 real patients’ specimens. The RGS3 was significantly highly expressed in tumor tissues (**P<0.01). (**C**) Statistic of the number of cases concerning the expression of RGS3 in HCC specimens. RGS3 is highly expressed in most of the tumor tissues (78/93) (*P*<0.01). (**D**) Representative graph of immunohistochemistry analysis (400×) of the HCC cases. The IgG antibody was used for staining the specimens as a control. RGS3 expression in tumor specimens was significantly higher than in adjacent non-cancerous tissues. (**E**) RGS3 shared a positively correlated with UBE2MP1 expression in the HCC tissues from our center, and the expression of RGS3 presented a remarkable decrease in HCC cells when UBE2MP1 was depleted (**P<0.01). (**F**) Predicted binding sequence of the 3’UTR of RGS3 mRNA with miR-145-5p. The Mfe value is calculated as: -28.2 kcal/mol. (**G**) The dual-luciferase reporter assay verified the direct interaction between miR-145-5p and RGS3 mRNA (**P<0.01).

Interestingly, we occasionally noticed that RGS3 is highly expressed in both HCC cell lines and HCC tissues (Figure 5A–5D). And notably, RGS3 shared a positively correlated with UBE2MP1 expression in the HCC tissues from our center, and the expression of RGS3 presented a remarkable decrease in HCC cells when UBE2MP1 was depleted (Figure 5E). We wondered if there exists a co-expression or regulation between UBE2MP1 and RGS3. On basis of this point, we tried to explore the role that miR-145-5p plays in this axis. The microcosm an online prediction software (https://www.microcosm.com/) indicated that miR-145-5p was an upstream regulator potentially interacting with the 3’-untranslated region (3’-UTR) of RGS3 mRNA with a high recommendation (Mfe: -28.2 kcal/mol) (Figure 5F). The dual-luciferase reporter assay was used for validating this assumpted direct binding between miR-145-5p and RGS3 mRNA. Similar to the above technique, we constructed the vectors containing a 202 bp sequence intercepted from the 3'UTR from the RGS3 mRNA (WT-UTR), and also the control luciferase vectors containing a mutated miR-145-5p targeting site (MUT-UTR). Both HepG2 and Hep3B cells were transfected with either the above two kinds of vectors and also mimicked by miR-145-5p. Here, taking HepG2 cells, for example, the miR-145-5p mimics (HepG2/miR-145-5p) significantly defected the luciferase signal of RGS3/pMIR/WT in comparison with the negative control (HepG2/NigmiR). The signal suppressive effect induced by miR-145-5p was abrogated in HepG2 cells transfected with a mutated miR-145-5p binding site (Figure 5G). And similar results were obtained from the Hep3B cell line too.

### Pseudogene UBE2MP1 enhances HCC tumor growth by modulating the miR-145-5p/RGS3 axis

**Figure 6:**
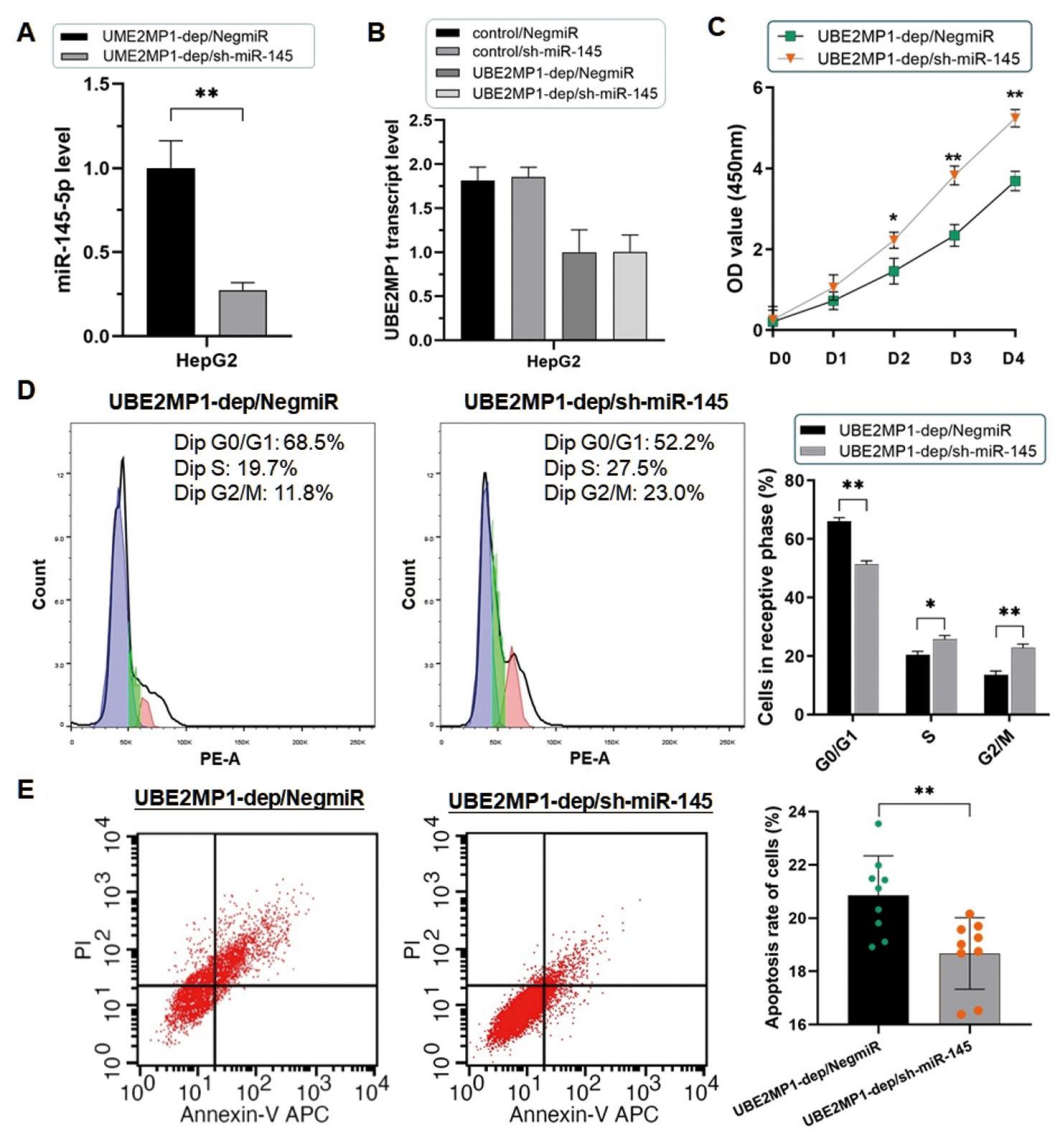
**Depletion of miR-145-5p rescued the phenotype induced by UBE2MP1 depletion.** (**A**) The RT-qPCR assay validated the effect of knocking out miR-145-5p in HepG2 cells (**P<0.01). (**B**) No significant change was induced on the UBE2MP1 transcript by miR-145-5p depleting. (**C**) The suppressed cell proliferation was significantly recovered by depleting miR-145-5p (*P<0.05, **P<0.01). (**D**) The arrested cell cycle induced by UBE2MP1 depletion in HepG2 cells was reversed when miR-145-5p was depleted (*P<0.05, **P<0.01). (**E**) The number of apoptotic cells was decreased in HepG2 cells with UBE2MP1 depletion when RGS3 was elevated (**P<0.01).

**Figure 7:**
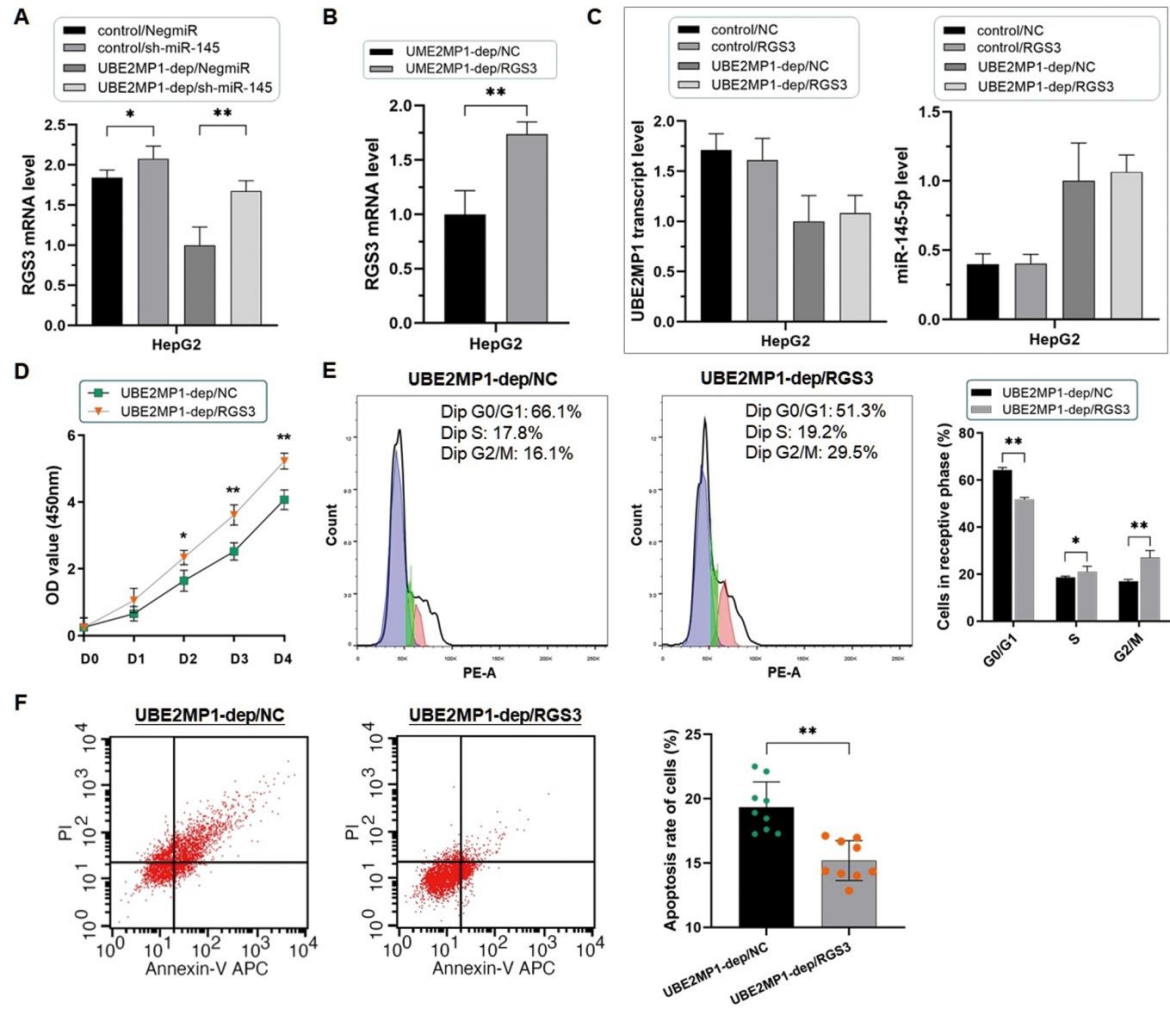
**Re-introducing RGS3 rescued the phenotype induced by UBE2MP1 depletion.** (**A**) Depletion of miR-145-5p significantly increases the expression of RGS3 in HepG2 cells (*P<0.05, **P<0.01). (**B**) Validation of the re-introduction of RGS3 in HepG2 cells treated with UBE2MP1 depletion (**P<0.01). (**C**) No significant changes were induced on miR-145-5p and UBE2MP1 transcript expression by re-introducing RGS3. (**D**, **E**) The suppressed cell proliferation was significantly recovered along with the escape of the G0/G1 stage arrest by re-introducing RGS3 (*P<0.05, **P<0.01). (**F**) The increased number of apoptotic cells declined in HepG2 cells with UBE2MP1 depletion when RGS3 was re-introduced (**P<0.01).

Since the verification of the post-transcriptional degeneration effect of miR-145-5p on RGS3 mRNA, we prompt that the phenotypes induced by UBE2MP1 in HCC are due to the modulation of the potential miR-145-5p/RGS3 axis. Take HepG2 cells as an example, firstly we lower the miR-145-5p expression in HepG2 cells treated with UBE2MP1 depletion again (Figure 6A). The suppressed cell proliferation was significantly recovered along with the escape of G0/G1 stage arrest (Figure 6B, 6C). Simultaneously, the number of apoptotic cells was decreased (Figure 6D). Secondly, we re-introduced RGS3, which had been down-regulated through UBE2MP1 depletion, in HepG2 cells. As expected, the knock-down of miR-145-5p led to an obvious increase in RGS3 expression at both mRNA and protein stages (Figure 7A). Interestingly, even though the expression of either UBE2MP1 or miR-145-5p was not impacted by re-introducing RGS3 in HCC cells, the cell proliferation and apoptosis statuses induced by UBE2MP1 depletion were effectively recovered RGS3 (Figure 7B–7F).

Last but not least, we simultaneously obtained similar results from the rescue experiments in Hep3B cell lines (Supplementary Figures 2, 3). All these findings above present an HCC-promoting axis of miR-145-5p/RGS3 under the control of UBE2MP1 through its ceRNA effect.

## DISCUSSION

HCC composes the majority portion of liver cancers and results in unsatisfactory outcomes and high mortality, due to the reckless growth and invasiveness, and potent tumoral heterogeneity [15, 16]. Recent strategies for targeting the particular genes in treating HCC patients systemically provide the researchers with a mighty approach to conquer the bottleneck of the rapid progress, high recurrence, and shorten overall survival of HCC. However, the therapeutic progress made so far is far from enough, and the comprehensive intracellular mechanisms promoting HCC leave us a lot for illustrating.

It has been a long time since the pseudogenes were regarded as ‘molecular fossils’ or ‘relics of evolution’ without definite intracellular functions [17]. However, anomalous transcription of the pseudogenes could be activated in the pathological situation and exerts particular effects on either oncogenic or tumor-suppressive events intracellular through different mechanisms [18, 19]. Recent evidence has gradually revealed the potential functions of pseudogene-derived transcripts, playing complex roles in the processes of transcription and post-transcription regulations and tumorigenesis and development [20].

In this study, we found that UBE2MP1 was activated to transcribe in HCC tissues, and mostly maintained at a high level, along with the common expression in the HCC cell lines much higher than the LO2 cells. UBE2M is the parental gene of UBE2MP1, and this gene encodes the protein as one of the key operators of the E2 ubiquitin-conjugating enzyme family [21]. As acknowledged, the E2 family is essential for the ubiquitination cascade, and UBE2M is a promoter of tumor onset and development, up-regulated in multiple human malignancies, including osteosarcoma, cholangiocarcinoma, and HCC [22, 23]. According to the detection of both the TCGA database and the real patients’ specimens, we validated the over-expression of UBE2M in HCC. Since the parental gene UBE2MP1 is a key mediator of ubiquitination, and has been reported as a tumor-promoter in HCC, we focused on this pseudogene and wondered if the abnormally transcribed UBE2MP1 participates in HCC cell growth dependently or independently to UBE2MP1. However, as a pseudogene of UBE2M, barely transcribed in the liver, there is no adequate information and report of its functions in cancer research.

The analysis of the real patients’ clinicopathological features demonstrated that the expression of UBE2MP1 is correlated with severe tumor progress and dismal phenotypes hindering a better prognosis and overall survival. We suppose that UBE2MP1 transcription participates in HCC progress through some mechanisms dependent or independent of its parental gene UBE2M. Based on the expression modulation of UBE2MP1 in HCC cell lines without inducing significant changes on UBE2M, we observed an efficient influence of either cell growth and maintenance when the UBE2MP1 transcript was depleted. The abrogation of UBE2MP1 not only suppressed tumor cell growth by arresting the cell cycle in the early stages, but also induced cell apoptosis. Obviously, the *in vitro* experiment indicated the independent effects of UBE2MP1 in the facilitation of HCC progress.

Wondering the exact mechanism of UBE2MP1 in promoting HCC growth, we investigated the possibility of the ceRNA effect implemented by its pseudogene-derived transcript. MiR-145-5p has been reported mainly to play the tumor-suppressive role in multiple malignancies. For instance, miR-145-5p induces tumor cell apoptosis in prostate cancer by degrading WIP1 [24]; MiR-145-5p is extremely decreased in breast cancer and attenuates paclitaxel resistance and suppresses the progression of breast cancer cells by targeting SOX2 [25]. For liver cancer, recent reports have noticed the tumor-suppressive effects of miR-145-5p in either cholangiocarcinoma or HCC by targeting different mRNAs, like CDCA3, and SPATS2, and involved in cell proliferation, apoptosis, or metastasis [26, 27]. Thus, we suppose that miR-145-5p is a key pivot of multiple regulating axes, impacting HCC progress and development through different mechanisms.

RGS3 gene encodes a regulator of the G-protein signaling (RGS) family and functions as a GTPase-activating protein for inhibiting G-protein-mediated signal transduction [28]. Accumulating evidence has indicated the pivotal function of RGS3 in participating in the Wnt signaling and the epithelial-mesenchymal transition, which strongly suggested RGS3 as a tumor enhancer. For example, RGS3 is highly expressed in gastric cancer and efficiently promotes tumor growth, and is correlated with poor prognosis [29]. And recently, RGS3 was reported to hinder the effect of KRASG12C inhibitors in the targeted therapy of lung cancer by enhancing the GTPase activity of KRAS [30]. In gastric cancer, RGS3 has been reported overexpressed in tumor cells and played a critical role in the Wnt signaling pathway on epithelial-mesenchymal transition [31]. For HCC, the limited reports suggested an enhancement of HCC cell apoptosis after its indirect down-regulation [32]. However, the definite function of RGS3 and the relative mechanism in HCC is not sufficient yet.

As mentioned in the introduction section, pseudogene transcripts may exert the ceRNA effect in tumor progression. For instance, the transcript of pseudogene BRAFP1 promotes lymphoma development by sponging a series of miRNAs, like miR-134 and miR-653, and efficiently preserves the expression of its parental gene BRAF and leads to the activation of the downstream MAPK pathway [33]; On the contrary, pseudogene PTENP1 exerts the ceRNA effects on the oncogenic miR-21 and miR-19, and sequentially protects the expression of its parental gene PTEN and assists to suppressive tumor development in gastric cancer and clear cell renal carcinoma [34, 35]. Simultaneously, Our recent study illustrated a ceRNA effect of pseudogene AKR1B10P1 transcript, sponging the tumor-suppressive miR-138 in HCC and resulting in an enhancement of tumor growth [36].

Similarly, in this study, we predicted the direct interaction of miR-145-5p with both RGS3 and the UBE2MP1 transcript at the same time. MiR-145-5p is a pivotal post-transcriptional regulator that has been described as a tumor suppressor in multiple human cancers. As reported, miR-145-5p was decreased to a low level in colon cancer, and elevation of miR-145-5p expression could significantly arrest the tumor cell cycle at the G0/G1 phase [37]. In liver cancer, miR-145-5p was reported as an inhibitor of cell proliferation by negatively targeting the oncogene Spermatogenesis associated serine-rich 2 (SPATS2) and Kruppel-like factor 5 (KLF5) [27, 38]. However, the further mechanism of miR-145-5p on HCC is in limitation, and we wonder if miR-145-5p is present to be the critical bridge between RGS3 and UBE2MP1. From this point, we separately discussed the existence of the degrading effect of miR-145-5p on RGS3 mRNA and the ceRNA effect between UBE2MP1 and miR-145-5p.

Firstly, the typical post-transcriptional regulation of miR-145-5p was validated through the dual-luciferase reporter assay and the 3’-UTR of RGS3 mRNA provides a particular site for binding to miR-145-5p. Since the complex network between the miRNAs and the verity of downstream mRNAs, we thought that the miR-145-5p/RGS3 axis might be one of the functional pathways affecting HCC progress and development. On the other hand, we discovered a specific binding site on the sequence of UBE2MP1 transcript for sponging miR-145-5p, and a remarkable increase of miR-145-5p in HCC cell lines through depleting UBE2MP1 supported the ceRNA effect of UBE2MP1 on defecting miR-145-5p and the tumor-suppressive effect. Moreover, the positive correlation between UBE2MP1 transcript and RGS3, and also the consequential strong decline of RGS3 through either UBE2MP1 depletion or elevation of miR-145-5p, definitely demonstrate RGS3 as a downstream effector controlled by UBE2MP1. Simultaneously, the impairment of cell growth and apoptosis resistance induced by UBE2MP1 depletion was significantly recovered by knocking down miR-145-5p or re-introducing RGS3. Thus, all these findings provide a convincible validation of the enhancement of HCC growth and maintenance of resisting cell apoptosis under the control of pseudogene UBE2MP1 through modulating the miR-145-5p/RGS3 axis.

In summary, our study discovered the transcriptional activation of pseudogene UBE2MP1 in HCC and validated the adverse correlation between UBE2MP1 transcript and outcomes in HCC patients. As the *in vitro* experiment demonstrated, the UBE2MP1 transcript potently facilitates HCC cell proliferation and apoptosis resistance, and the ceRNA effect of it provides a critical mechanism of UBE2MP1, independent of its parental gene, in facilitating HCC cell growth and maintenance via modulating the miR-145-5p/RGS3 axis. However, limited by lacking the *in vivo* experiment, it still needs further exploration of the exact function of the UBE2MP1 transcript in the animal models, and we intend to carry out the orthotopic transplantation of mouse liver to give out more convincible evidence to support our findings. And lastly, we suppose that UBE2MP1 and its downstream miR-145-5p/RGS3 axis might be the potential and hopeful targets for HCC prevention and therapeutic strategy, even though there is still much detail for investigation.

## Supplementary Material

Supplementary Figures

Supplementary Tables

## References

[1] Piñero F, Dirchwolf M, Pessôa MG. Biomarkers in Hepatocellular Carcinoma: Diagnosis, Prognosis and Treatment Response Assessment. Cells. 2020; 9:1370. 10.3390/cells9061370 32492896 PMC7349517

[2] Llovet JM, Kelley RK, Villanueva A, Singal AG, Pikarsky E, Roayaie S, Lencioni R, Koike K, Zucman-Rossi J, Finn RS. Hepatocellular carcinoma. Nat Rev Dis Primers. 2021; 7:6. 10.1038/s41572-020-00240-3 33479224 PMC13537433

[3] Siegel RL, Miller KD, Jemal A. Cancer statistics, 2019. CA Cancer J Clin. 2019; 69:7–34. 10.3322/caac.21551 30620402

[4] Peng WX, Koirala P, Mo YY. LncRNA-mediated regulation of cell signaling in cancer. Oncogene. 2017; 36:5661–7. 10.1038/onc.2017.184 28604750 PMC6450570

[5] Chan JJ, Tay Y. Noncoding RNA:RNA Regulatory Networks in Cancer. Int J Mol Sci. 2018; 19:1310. 10.3390/ijms19051310 29702599 PMC5983611

[6] Uszczynska-Ratajczak B, Lagarde J, Frankish A, Guigó R, Johnson R. Towards a complete map of the human long non-coding RNA transcriptome. Nat Rev Genet. 2018; 19:535–48. 10.1038/s41576-018-0017-y 29795125 PMC6451964

[7] Statello L, Guo CJ, Chen LL, Huarte M. Gene regulation by long non-coding RNAs and its biological functions. Nat Rev Mol Cell Biol. 2021; 22:96–118. 10.1038/s41580-020-00315-9 33353982 PMC7754182

[8] Lister NC, Johnsson P, Waters PD, Morris KV. Pseudogenes: A Novel Source of Trans-Acting Antisense RNAs. Methods Mol Biol. 2021; 2324:219–36. 10.1007/978-1-0716-1503-4_14 34165718

[9] Kim MS, Pinto SM, Getnet D, Nirujogi RS, Manda SS, Chaerkady R, Madugundu AK, Kelkar DS, Isserlin R, Jain S, Thomas JK, Muthusamy B, Leal-Rojas P, et al. A draft map of the human proteome. Nature. 2014; 509:575–81. 10.1038/nature13302 24870542 PMC4403737

[10] Sasidharan R, Gerstein M. Genomics: protein fossils live on as RNA. Nature. 2008; 453:729–31. 10.1038/453729a 18528383

[11] Harper KL, Mottram TJ, Whitehouse A. Insights into the Evolving Roles of Circular RNAs in Cancer. Cancers (Basel). 2021; 13:4180. 10.3390/cancers13164180 34439334 PMC8391132

[12] Xie C, Zhang LZ, Chen ZL, Zhong WJ, Fang JH, Zhu Y, Xiao MH, Guo ZW, Zhao N, He X, Zhuang SM. A hMTR4-PDIA3P1-miR-125/124-TRAF6 Regulatory Axis and Its Function in NF kappa B Signaling and Chemoresistance. Hepatology. 2020; 71:1660–77. 10.1002/hep.30931 31509261 PMC7318625

[13] Wang J, Wang X, Bhat A, Chen Y, Xu K, Mo YY, Yi SS, Zhou Y. Comprehensive Network Analysis Reveals Alternative Splicing-Related lncRNAs in Hepatocellular Carcinoma. Front Genet. 2020; 11:659. 10.3389/fgene.2020.00659 32760422 PMC7373802

[14] Wang N, Hao F, Ren J, Fei X, Chen Y, Xu W, Wang J. Positive feedback loop of AKR1B10P1/miR-138/SOX4 promotes cell growth in hepatocellular carcinoma cells. Am J Transl Res. 2020; 12:5465–80. 33042431 PMC7540089

[15] Wong MC, Jiang JY, Goggins WB, Liang M, Fang Y, Fung FD, Leung C, Wang HH, Wong GL, Wong VW, Chan HL. International incidence and mortality trends of liver cancer: a global profile. Sci Rep. 2017; 7:45846. 10.1038/srep45846 28361988 PMC5374459

[16] Llovet JM, Zucman-Rossi J, Pikarsky E, Sangro B, Schwartz M, Sherman M, Gores G. Hepatocellular carcinoma. Nat Rev Dis Primers. 2016; 2:16018. 10.1038/nrdp.2016.18 27158749

[17] Zhu Y, Orre LM, Johansson HJ, Huss M, Boekel J, Vesterlund M, Fernandez-Woodbridge A, Branca RM, Lehtiö J. Discovery of coding regions in the human genome by integrated proteogenomics analysis workflow. Nat Commun. 2018; 9:903. 10.1038/s41467-018-03311-y 29500430 PMC5834625

[18] Wang K, Sun Y, Guo C, Liu T, Fei X, Chang C. Androgen receptor regulates ASS1P3/miR-34a-5p/ASS1 signaling to promote renal cell carcinoma cell growth. Cell Death Dis. 2019; 10:339. 10.1038/s41419-019-1330-x 31000693 PMC6472417

[19] Thomson DW, Dinger ME. Endogenous microRNA sponges: evidence and controversy. Nat Rev Genet. 2016; 17:272–83. 10.1038/nrg.2016.20 27040487

[20] Singh RK, Singh D, Yadava A, Srivastava AK. Molecular fossils “pseudogenes” as functional signature in biological system. Genes Genomics. 2020; 42:619–30. 10.1007/s13258-020-00935-7 32277362

[21] Li X, Elmira E, Rohondia S, Wang J, Liu J, Dou QP. A patent review of the ubiquitin ligase system: 2015-2018. Expert Opin Ther Pat. 2018; 28:919–37. 10.1080/13543776.2018.1549229 30449221 PMC6398165

[22] Zhang Y, Shi CC, Zhang HP, Li GQ, Li SS. MLN4924 suppresses neddylation and induces cell cycle arrest, senescence, and apoptosis in human osteosarcoma. Oncotarget. 2016; 7:45263–74. 10.18632/oncotarget.9481 27223074 PMC5216721

[23] Zhang GC, Yu XN, Sun JL, Xiong J, Yang YJ, Jiang XM, Zhu JM. UBE2M promotes cell proliferation via the β-catenin/cyclin D1 signaling in hepatocellular carcinoma. Aging (Albany NY). 2020; 12:2373–92. 10.18632/aging.102749 32012120 PMC7041726

[24] Sun J, Deng L, Gong Y. MiR-145-5p Inhibits the Invasion of Prostate Cancer and Induces Apoptosis by Inhibiting WIP1. J Oncol. 2021; 2021:4412705. 10.1155/2021/4412705 34899906 PMC8660234

[25] Guan X, Guan Y. miR-145-5p attenuates paclitaxel resistance and suppresses the progression in drug-resistant breast cancer cell lines. Neoplasma. 2020; 67:972–81. 10.4149/neo_2020_190622N536 32412771

[26] Gu X, Zhang J, Ran Y, Pan H, Jia J, Zhao Y, Zhao X, Li W, Song S, Yu X. Circular RNA hsa_circ_101555 promotes hepatocellular carcinoma cell proliferation and migration by sponging miR-145-5p and regulating CDCA3 expression. Cell Death Dis. 2021; 12:356. 10.1038/s41419-021-03626-7 33824281 PMC8024300

[27] Dong G, Zhang S, Shen S, Sun L, Wang X, Wang H, Wu J, Liu T, Wang C, Wang H, Lu T, Rao B, Ren Z. SPATS2, negatively regulated by miR-145-5p, promotes hepatocellular carcinoma progression through regulating cell cycle. Cell Death Dis. 2020; 11:837. 10.1038/s41419-020-03039-y 33037180 PMC7547105

[28] Sethakorn N, Dulin NO. RGS expression in cancer: oncomining the cancer microarray data. J Recept Signal Transduct Res. 2013; 33:166–71. 10.3109/10799893.2013.773450 23464602

[29] Wang J, Zhou Y, Fei X, Chen X, Zhu Z. Regulator of G-protein signaling 3 targeted by miR-126 correlates with poor prognosis in gastric cancer patients. Anticancer Drugs. 2017; 28:161–9. 10.1097/CAD.0000000000000446 27754994

[30] Li C, Vides A, Kim D, Xue JY, Zhao Y, Lito P. The G protein signaling regulator RGS3 enhances the GTPase activity of KRAS. Science. 2021; 374:197–201. 10.1126/science.abf1730 34618566 PMC9295010

[31] Li W, Si X, Yang J, Zhang J, Yu K, Cao Y. Regulator of G-protein signalling 3 and its regulator microRNA-133a mediate cell proliferation in gastric cancer. Arab J Gastroenterol. 2020; 21:237–45. 10.1016/j.ajg.2020.07.011 32928707

[32] Lu S, Zhou J, Sun Y, Li N, Miao M, Jiao B, Chen H. The noncoding RNA HOXD-AS1 is a critical regulator of the metastasis and apoptosis phenotype in human hepatocellular carcinoma. Mol Cancer. 2017; 16:125. 10.1186/s12943-017-0676-x 28724429 PMC5518122

[33] Karreth FA, Reschke M, Ruocco A, Ng C, Chapuy B, Léopold V, Sjoberg M, Keane TM, Verma A, Ala U, Tay Y, Wu D, Seitzer N, et al. The BRAF pseudogene functions as a competitive endogenous RNA and induces lymphoma *in vivo*. Cell. 2015; 161:319–32. 10.1016/j.cell.2015.02.043 25843629 PMC6922011

[34] Poliseno L, Salmena L, Zhang J, Carver B, Haveman WJ, Pandolfi PP. A coding-independent function of gene and pseudogene mRNAs regulates tumour biology. Nature. 2010; 465:1033–8. 10.1038/nature09144 20577206 PMC3206313

[35] Zhang R, Guo Y, Ma Z, Ma G, Xue Q, Li F, Liu L. Long non-coding RNA PTENP1 functions as a ceRNA to modulate PTEN level by decoying miR-106b and miR-93 in gastric cancer. Oncotarget. 2017; 8:26079–89. 10.18632/oncotarget.15317 28212532 PMC5432239

[36] Hao F, Fei X, Ren X, Xi Xiao J, Chen Y, Wang J. Pseudogene AKR1B10P1 enhances tumorigenicity and regulates epithelial-mesenchymal transition in hepatocellular carcinoma via stabilizing SOX4. J Cell Mol Med. 2020; 24:11779–90. 10.1111/jcmm.15790 32924268 PMC7579691

[37] Chen Q, Zhou L, Ye X, Tao M, Wu J. miR-145-5p suppresses proliferation, metastasis and EMT of colorectal cancer by targeting CDCA3. Pathol Res Pract. 2020; 216:152872. 10.1016/j.prp.2020.152872 32107086

[38] Liang H, Sun H, Yang J, Yi C. miR-145-5p reduces proliferation and migration of hepatocellular carcinoma by targeting KLF5. Mol Med Rep. 2018; 17:8332–8. 10.3892/mmr.2018.8880 29658584

