# Supplementary material for: Pseudogene UBE2MP1 derived transcript enhances in vitro cell proliferation and apoptosis resistance of hepatocellular carcinoma cells through miR-145-5p/RGS3 axis
Source: Aging (Albany NY). 2022 Oct 7;14(19):7906–25. doi: 10.18632/aging.204319 (PMC9596209; doi:10.18632/aging.204319)
Supplement: Supplementary Figures [file aging-14-204319-s001.pdf]

SUPPLEMENTARY FIGURES

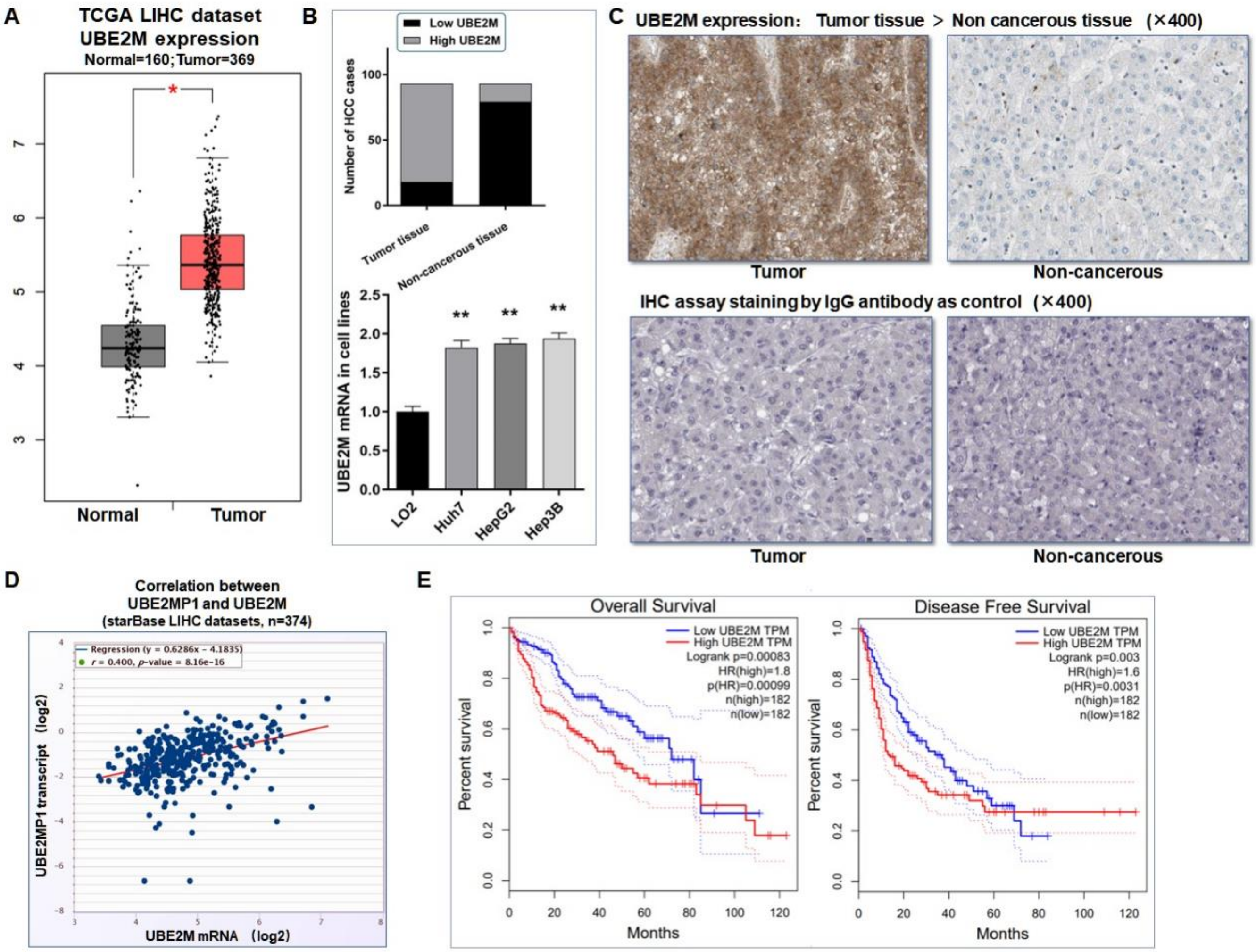

Supplementary Figure 1. The expression profile of the parental gene UBE2M in HCC. (**A**) UBE2M expression profile in HCC from the TCGA database (**P*<0.05). (**B**) UBE2M was significantly highly expressed in HCC tissues and cell lines (***P*<0.01). (**C**) Representative graph of immunohistochemistry analysis (400×) of the HCC cases. UBE2M expression in tumor specimens was significantly higher than in adjacent non-cancerous tissues. (**D**) UBE2M and the pseudogene UBE2MP1 are correlated in expression with high significance. (**E**) High expression of UBE2M in tumor tissues is related to shortened overall survival (OS) and disease-free survival (DFS) in HCC patients.

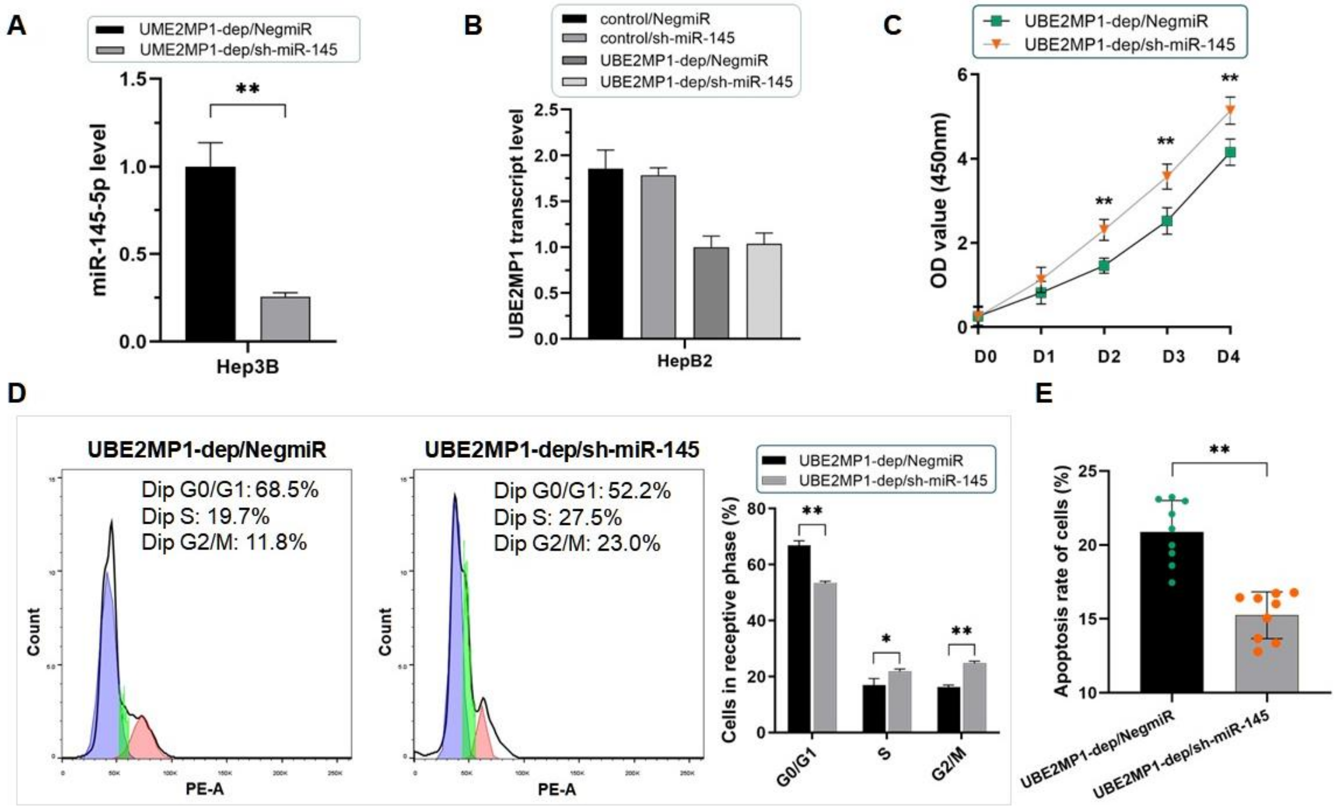

Supplementary Figure 2. Depletion of miR-145-5p in Hep3B cells rescued the phenotype induced by UBE2MP1 depletion. (**A**) The RT-qPCR assay validated the effect of knocking out miR-145-5p in Hep3B cells (***P*<0.01). (**B**) No significant change was induced on the UBE2MP1 transcript by miR-145-5p depleting. (**C**) The suppressed cell proliferation was significantly recovered by depleting miR-145-5p (***P*<0.01). (**D**) The arrested cell cycle induced by UBE2MP1 depletion in Hep3B cells was reversed when miR-145-5p was depleted. The percentage of cells in the G0/G1 phase decreased from 66.80% to 53.37% (*P*<0.01). The percentage decrease was observed in the S phase (from 16.97% to 21.80%, *P*<0.05) and the G2/M phase (from 16.23% to 24.83%, *P*<0.01). (**E**) The number of apoptotic cells was decreased in Hep3B cells with UBE2MP1 depletion when RGS3 was elevated (declined from 20.52% to 13.65%, ***P*<0.01).

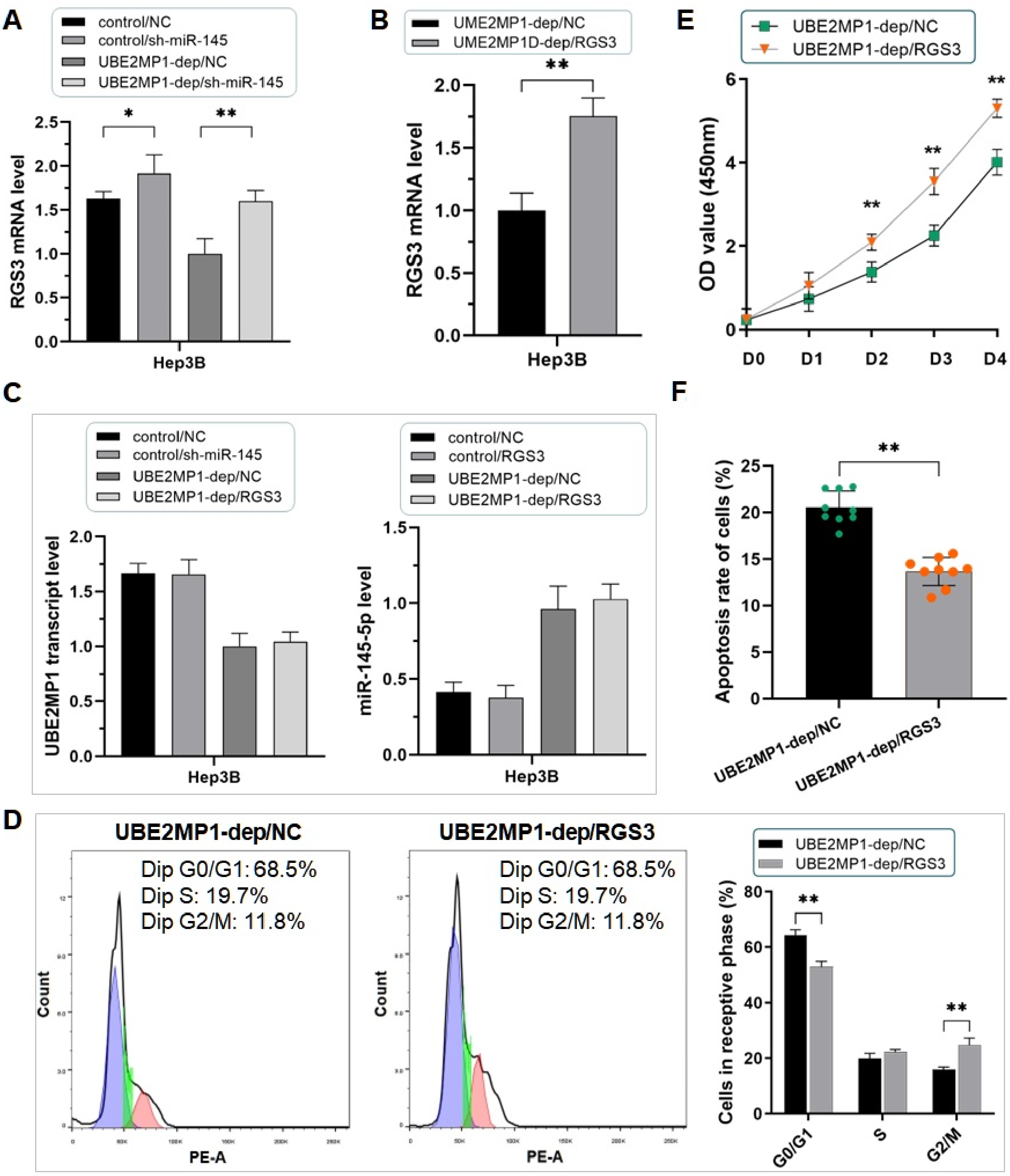

Supplementary Figure 3. Re-introducing RGS3 in Hep3B cells rescued the phenotype induced by UBE2MP1 depletion. (**A**) Depletion of miR-145-5p significantly increases the expression of RGS3 in Hep3B cells (**P*<0.05, ***P*<0.01). (**B**) Validation of the re-introduction of RGS3 in Hep3B cells treated with UBE2MP1 depletion (***P*<0.01). (**C**) No significant changes were induced on miR-145-5p and UBE2MP1 transcript expression by re-introducing RGS3. (**D**, **E**). The suppressed cell proliferation was significantly recovered along with the escape of the G0/G1 stage arrest by re-introducing RGS3 (**P*<0.05, ***P*<0.01). (**F**) The increased number of apoptotic cells declined in Hep3B cells with UBE2MP1 depletion when RGS3 was re-introduced (***P*<0.01).
