# Supplementary material for: Pseudogene UBE2MP1 derived transcript enhances in vitro cell proliferation and apoptosis resistance of hepatocellular carcinoma cells through miR-145-5p/RGS3 axis
Source: Aging (Albany NY). 2022 Oct 7;14(19):7906–25. doi: 10.18632/aging.204319 (PMC9596209; doi:10.18632/aging.204319)
Supplement: Supplementary Tables [file aging-14-204319-s002.pdf]

SUPPLEMENTARY TABLES

Supplementary Table 1. The primers for RT-qPCR assay.

Genes	Forward	Reverse
UBE2MP1	5’-AGATCCAGACGACCTCCTCAA-3’	5’-CCATCCCGCATGGATGGCT-3’
UBE2M	5’- CCTGCCCAAGACGTGTGATA -3’	5’- CCCTGGCCCACCTTAAAACT -3’
miR-145-5p	5’-CTCAACTGGTGTCGTGGATCCCTAAG-3’	5’-ACACTCCAGCTGGGGTCCAGTTTTCCCAG-3’

Supplementary Table 2. The selected sequences of the predicted miR-145-5p binding site of the UBE2MP1 transcript and the 3’-UTR of RGS3 mRNA, along with the relative mutated sequences.

Genes	Sequence including the binding site (202 bp)	Relative mutated sequence
UBE2MP1	5’-ccagggttacccgcatgatcccccccaaggtgaagtgtgagacaatggtctatcaccccaacattgaaatcgagggcaacgtctgcctcaacatcctcagagagaactggaagccagtccttacgataaactccataatttatggcctgcagtatctctacttggagcccaaccccgaggacccactgaacaagaaggccgca-3’	5’-gacgctaagcgggaagttgcgcgcaacgagtactctcacagattcgacaaactcgcgatcttagtattggtgcggatcctgtccgtgatcttgcactgtgtgtagtggtaccgactgcattccaaatagtgctttaataaagccgtccagaaacacaagtagcaccgctagcgccacgtcgctcagtagatgtagggccctc-3’
3’-UTR of RGS3 mRNA	5’-tcgtcccgggcaccggtggcaggcagctggccttctggactaaggcagcctgggggggacactgcagtctggctacacacagagatctggcaccccctgggtggagtgtccctcgggggctttgggaaagcatggcacccctcagaccacacagtagccaagttctggagcaaataaaaggcctgtgttatttcttgttcttga-3’	5’-acctcccgcggagccgagcctgcctggtgggcatgtcgtcaatgcctggcagcgcgcagagtcctgacagccaagagagacacaacagcctcgcgcagcgagcactctgcgtggcgcggtatcgcataccttcggagcgtgacagctctctgaaccgatgatgtcgtggataaatatgccgtctctaaatacatctacatca-3’
